# Value of different anastomoses in laparoscopic radical right hemicolectomy for right-sided colon cancer: retrospective study and literature review

**DOI:** 10.1186/s12957-022-02789-7

**Published:** 2022-09-29

**Authors:** Xiaoming Wang, Hongyan Ni, Wangqiang Jia, Sen Wang, Yangyang Zhang, Peng Zhao, Long Yuan

**Affiliations:** 1 Department of Surgery, The Affiliated Cancer Hospital of Zhengzhou University & Henan Cancer Hospital, Zhengzhou, 450008 China; 2 Department of Surgery, Henan No.3 Provincial People’s Hospital, Zhengzhou, 450008 China; 3 Department of Tumor Surgery, The First Hospital of Lanzhou University & The First Clinical Medical College of Lanzhou University, Lanzhou, 730000 China

**Keywords:** Right colon cancer, Colon tumor, Lateral anastomosis, Anastomotic leak

## Abstract

**Background:**

This study aimed to analyze the safety of circular lateral anastomosis and cross-lateral anastomosis in laparoscopic radical resection of right-sided colon cancer.

**Methods:**

From January 2018 to March 2021, 147 patients with right-sided colon cancer were admitted to the Department of General Surgery, Cancer Hospital, Zhengzhou University. The experimental group comprised patients with circular lateral anastomosis, whereas the control group comprised patients with cruciform lateral anastomosis. The general clinical data, intraoperative features, and postoperative results of the two groups were compared and analyzed.

**Results:**

Both groups successfully underwent laparoscopic lateral ileocolic anastomosis, with significant differences in anastomotic leakage (*χ*^2^=4.520, *P* < 0.05). By contrast, body mass index (t = 1.568, *P* = 0.119), histological typing (*χ*^2^ = 2.067, *P* = 0.559), intraoperative bleeding (t = 0.418, *P* = 0.677), and intestinal obstruction (*χ*^2^ = 2.564, *P* = 0.109) were not significantly different between the groups (*P* > 0.05).

**Conclusions:**

In laparoscopic-assisted radical hemicolectomy for right-sided colon cancer, the incidence of postoperative anastomotic leakage was lower with circular lateral anastomosis than with cross-lateral anastomosis, and circular lateral anastomosis was superior to cross-lateral anastomosis in terms of reducing the length of hospital stay and improving patients' postoperative quality of life.

## Background

Colon cancer is one of the most common malignant tumors of the gastrointestinal system, and colorectal cancer is the third most common among men and the second most common among women [1]. Right hemicolectomy accounts for approximately 42% of colorectal cancers [2]. Radical hemicolectomy with laparoscopic assistance is a well-established procedure for right-sided colon cancer [3]. It has gradually replaced traditional open surgery as the primary treatment modality for right hemicolectomy of colon cancer [4, 5] because it results in minor trauma, causes less bleeding, and has a faster postoperative recovery [6–8]. In radical surgery for right hemicolectomy, anastomoses of the ileum are various, and lateral anastomosis is common [9, 10]. After resection when performing right hemicolectomy, different anastomoses will affect anastomotic blood flow and tension and become a risk factor for postoperative anastomotic leakage [11]. Because the anastomosis is located at the level of the transverse colon in the upper abdomen, if anastomotic leakage occurs, severe infection may occur in the entire abdominal cavity and intestinal adhesions, intestinal obstruction, and other comorbidities may likely develop, which may even lead to perioperative death in serious cases. This study aimed to provide a new basis for intraoperative anastomosis in patients who undergo right hemicolectomy by comparing the safety of circular lateral anastomosis with that of cross-lateral anastomosis used in laparoscopic radical right hemicolectomy.

## Methods

### Clinical information

In total, 147 patients who underwent laparoscopic-assisted radical right hemicolectomy for right-sided colon cancer in the Department of General Surgery of the Cancer Hospital of Zhengzhou University between January 2018 and March 2021 were included in the study. This study enrolled patients with standard right-sided colon cancer. The tumors were located in the ileocecal region, ascending colon, and near the hepatic flexure of the colon. Preoperative examination and intraoperative exploration were needed to observe the invasion of the surrounding organs during the surgical treatment of tumors located in the hepatic region of the colon. The inclusion criteria were as follows: right hemicolectomy and pathological biopsy confirmed the diagnosis of right hemicolectomized colon cancer; with feasible radical tumor resection based on enhanced CT of the abdomen and intestinal angiography; underwent preoperative MRI or enhanced CT to assess regional lymph node involvement and exclude the presence of metastases; and underwent preoperative multidisciplinary consultation (multidisciplinary team, MDT) and recommended for surgical treatment. Meanwhile, the exclusion criteria were as follows: requiring extended right hemicolectomy; with severe systemic diseases that cannot tolerate surgery, such as impaired heart, kidney, liver, and lung functions; laparoscopic exploration revealed extensive intra-abdominal adhesions that were difficult to separate laparoscopically and required intermediate open surgery; with tumors involving a large area, peritoneal metastasis, extensive lymph node metastasis, or tumor encircling essential blood vessels; and with advanced cancer and poor overall physical condition that cannot tolerate surgery and requires systemic comprehensive treatment. The tumor–node–metastasis staging system of the American Joint Commission on Cancer based on tumor size or extent, lymph node involvement, and distant metastasis was considered [12]. All the patients and their families provided their written informed consent. All the study protocols complied with the 1975 Declaration of Helsinki.

### Surgical procedure

**Fig. 1:**
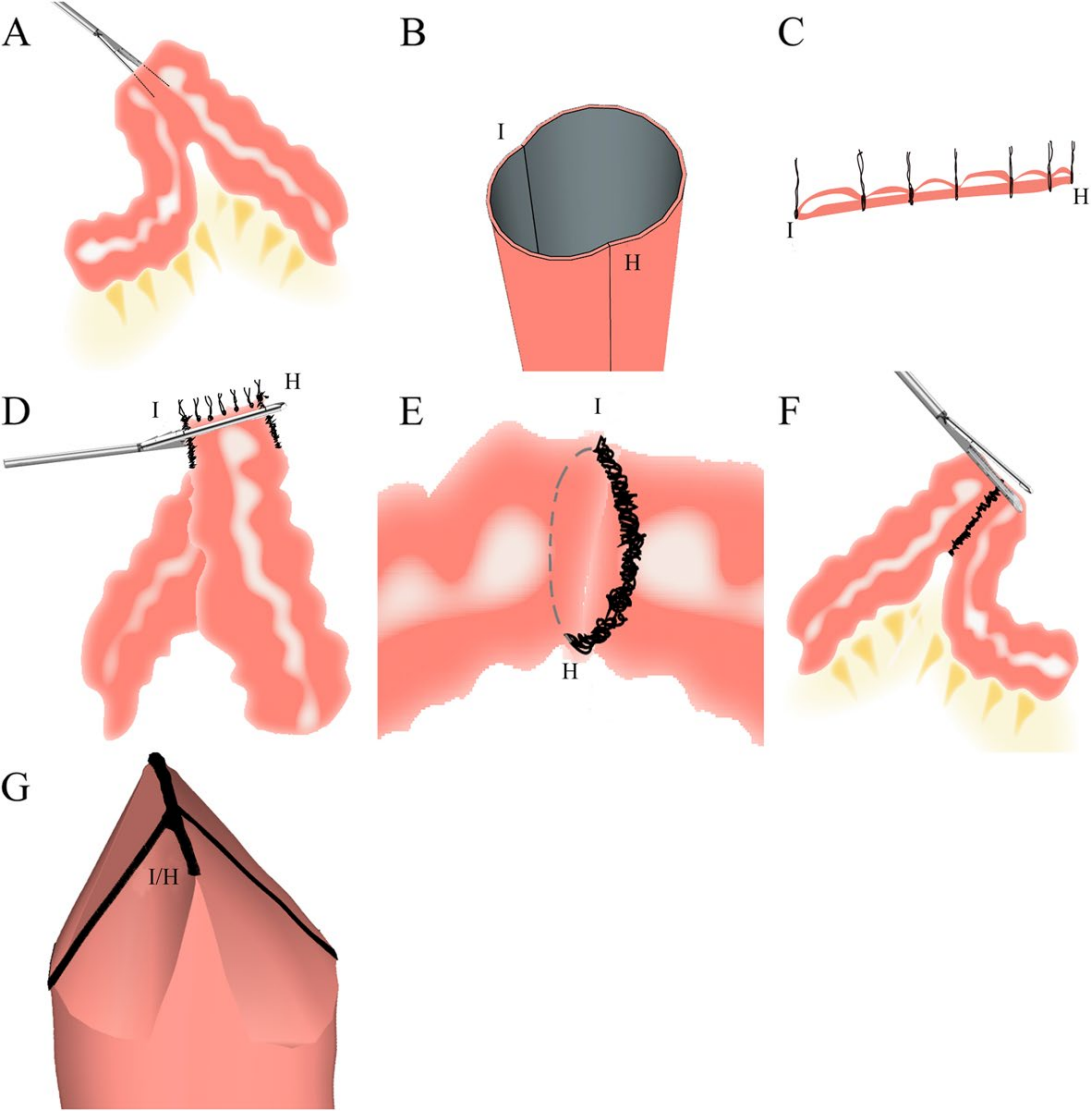
The procedure of two different anastomosis methods for right-sided colon cancer. **A** After excision of the mass, the small intestine and colonic stump were closed with a stapler. **B** The common opening formed by the two intestinal sites after closure, I and H, are the meeting points of the junction between the anastomotic nail and the stump. **C** To ensure a straight line between I and H points when closing the common stump of ileocolonic, and I and H are the starting and ending points, the interrupted suture method (approximately 5–7 stitches for lifting and fixation) is used between the starting and ending points. **D** All sutures were lifted, and the common stump was closed with a disposable linear stapler, with the common opening forming a horizontal line. **E** After closing the common opening, the anastomosis is annular at this time. **F** I and H points in the control group were close to or overlapped when the ileocolonic lateral anastomosis was performed. **G** After the closure of the common stump in the control group, the anastomosis had a cruciform shape

All patients underwent a systematic routine preoperative examination and clinical analysis; they were evaluated by an MDT, had indications for surgery, and had no contraindications. The same surgeon and fixed assistant performed all the operations. The procedure was divided into two stages. The first stage was lateral anastomosis, in which the patient was in supine with the legs separated. After endotracheal intubation and induction of general anesthesia, a routine disinfection towel was placed. A pneumoperitoneum needle was inserted 5 cm below the umbilicus to establish pneumoperitoneum, and the intra-abdominal pressure was maintained between 12 and 15 mmHg. A trocar was placed, laparoscopy was performed to explore the abdominal cavity, and each surgical hole was opened successively. The superior mesenteric vein and ileocolic vessels were fully exposed, Toldt’s space was entered, the root of the ileocolic vessels was revealed by freeing to the outer edge of the right-sided colon, and the ileocolic arterioles were severed and ligated. The right colonic artery and vein were exposed and disconnected, the transverse colonic mesentery was opened, and the right branches of the colonic vessels were disconnected and ligated. The greater omentum and gastrocolic ligament were opened to pass through the Toldt's gap along the root of the ileocecal mesentery to the lower edge of the horizontal portion of the duodenum. The pneumoperitoneum and each trocar were removed, a 5-cm incision was made in the middle of the umbilicus and covered with an incision protector, and the tumor was removed. The ileum was then dissected 10 cm from the ileocecal region, and the transverse colon was analyzed in the middle of the transverse colon. The intestinal blood supply was evaluated by cutting a thin layer of intestinal serosa with scissors and observing bleeding at the cutting site based on laparoscopic colorectal cancer resection scope guidelines [13–15]. After resection when performing the right hemicolectomy, a disposable linear cutting closure device (Purun Purple nail tank, 75 mm) was placed into the ileocecal stump and closed at the contralateral edge of the ileum and colonic mesentery, at which time the ileocecal stump formed a typical opening (Fig. 1A, B). At this point, the first stage of the lateral anastomosis was completed. The second stage is closure of the typical stump, in which the standard opening formed by the ileocolic stump is used in circular lateral anastomosis. The “1” absorbable line can suspend and straighten the two points I and H. Because the diameter of the colon is more significant than that of the ileum in normal anatomy, to ensure a straight line between points I and H, which are the starting and ending points, respectively, interrupted sutures are used between the starting and ending points (approximately 5–7 stitches for lifting and fixing) when closing the familiar stump of the ileum (Fig. 1C). All sutures were lifted, and the familiar stump was closed with a disposable linear cut closure, with the standard opening in a horizontal line, where the anastomosis was circular (Fig. 1D–E). In the control group, a crossed lateral anastomosis was used, in which points I and H were brought close together or overlapped during the lateral ileocolic anastomosis, and the stump was closed with a disposable linear cut closure, at which point the anastomosis was cross-shaped (Fig. 1F–G). The anastomotic staples in both groups were intermittently reinforced with a “1” absorbable thread. After closure of the common anastomosis, the intestinal color, mesangial blood supply, intestinal peristalsis, and other conditions were observed to evaluate the anastomosis to ensure adequate blood supply and patency of the anastomosis. Patients who underwent circular lateral anastomosis were considered the experimental group and those who underwent cross-shaped lateral anastomosis were considered the control group.

### Observed indicators

Intraoperative data included operation time, intraoperative blood loss, American Society of Anesthesiologists (ASA) score, and anastomosis time. Postoperative variables included length of postoperative hospitalization, first postoperative feeding, first defecation, first postoperative routine blood test (albumin, hemoglobin, red blood cells), and postoperative complications (intestinal obstruction, anastomotic leak, anastomotic stricture, and anastomotic bleeding).

### Statistical methods

The statistical software SPSS version 26.0 was used for computational analysis, and normally distributed measures are expressed as mean ± standard deviation (mean ± SD). An independent samples *t*-test was used for comparisons between groups. Count data are expressed as *n* (%), and comparisons of count variables between groups were analyzed using the *χ*^2^ test. If the sample size was small or the expected value of a cell was < 5, the likelihood ratio *χ*^2^ test was used. Differences were considered significant at *P* < 0.05.

## Results

### Comparison of patients' preoperative general information

In total, 147 patients were enrolled in this study, with 77 and 70 patients in the experimental and control groups, respectively. The results were analyzed after grouping. The experimental group comprised 42 male and 35 female patients, with a maximum age of 87 years, minimum age of 28 years, and an average age of 58.05 ± 1.45 years. Meanwhile, the control group comprised 41 male and 29 female patients with a maximum age of 81 years, minimum age of 31 years, and average age of 59.36 ± 1.34 years. The differences between the two groups were not significant (*P* > 0.05) and were comparable in terms of sex, age, body mass index, neoadjuvant therapy, right hemicolectomy tumor site, and underlying diseases (Table 1).

**Table 1:** Preoperative basic data analysis

Variable	Control group (*n* = 70)	Experimental group (*n* = 77)	Statistical data	*P*-value
Sex^a^ (*n*)			0.242	0.623
Male	41	42		
Female	29	35		
Age^b^ (years)	59.36 ± 1.34	58.05 ± 1.45	−0.658	0.511
BMI^b^ (kg/m^2^, mean ± SD)	23.06 ± 0.30	23.71 ± 0.29	1.568	0.119
Neoadjuvant therapy^a^ (*n*)			1.901	0.168
Yes	8	4		
No	62	73		
Tumor site^a^ (*n*)			1.851	0.174
Ileocecal region	55	67		
Near the liver area	15	10		
Tumor size (cm)^b^ (*n*)	5.68 ± 1.08	5.49 ± 0.88	−0.97	0.334
AJCC stage (TNM)^a^ (*n*)			1.136	0.567
Period I	5	8		
Period II	37	44		
Period III	28	25		
Preoperative medication^a^ (*n*)			1.009	0.604
Anticoagulants	11	9		
Steroid drugs	2	1		
Other (including none)	57	67		
Underlying diseases^a^ (*n*)				
Hypertension	17	16	0.259	0.611
Diabetes	10	7	0.968	0.325
Heart disease	6	12	1.678	0.195
Cerebral infarction	7	8	0.006	0.938

Control group, cross-lateral anastomosis; experimental group, circular side-to-side anastomosis; *SD* standard deviation, *AJCC* American Joint Commission on Cancer, *TNM* tumor–node–metastasis, *BMI* body mass index

^a^Chi-square test

^b^*t*-test

### Comparison of intraoperative observation indices

Patients in the experimental and control groups underwent radical right hemicolectomy for right-sided colon cancer, and an R0 resection was achieved in all the patients. The total operation time was 207.27 ± 66.28 min in the experimental group and 182.44 ± 64.61 min in the control group (*t* = 2.296, *P* = 0.859), and the operation time of the cross-lateral anastomosis was less than that of the circular lateral anastomosis. The mean intraoperative blood loss was 156.36 ± 100.46 ml in the experimental group and 150 ± 82.09 ml in the control group (*t* = 0.418, *P* = 0.677). The mean ASA score was 2.03 ± 0.43 in the experimental group and 1.97 ± 0.48 in the control group (*t* = 0.727, *P* = 0.468). The analysis revealed that the differences in operation time, intraoperative bleeding, and ASA were not significant between the two groups (*P* > 0.05). The results are presented in Table 2.

**Table 2:** Intraoperative factor analysis

	Control group (*n* = 70)	Experimental group (*n* = 77)	Statistical data	*P*-value
Operation time^a^ (min)	182.44 ± 64.61	207.27 ± 66.28	2.296	0.859
Intraoperative bleeding^a^ (ml)	150 ± 82.09	156.36 ± 100.46	0.418	0.677
ASA^a^	1.97 ± 0.48	2.03 ± 0.43	0.727	0.468

*ASA* American Society of Anesthesiologists; control group, cross-lateral anastomosis; experimental group, circular lateral anastomosis

^a^*t*-test

### Comparison of postoperative observation indicators

According to the postoperative observation index, one patient (1.3%) in the experimental group and two (2.8 %) in the control group had anastomotic bleeding. All three patients were cured with hemostatic drugs and water fasting after conservative treatment. Among the postoperative complications, one patient (1.3%) in the experimental group and one (1.4%) in the control group had anastomotic stenosis. Both patients had mild anastomotic stenosis, as suggested by imaging, and the patients did not complain of significant discomfort and were not treated. One patient (1.3%) in the experimental group and two patients (2.9 %) in the control group had intestinal obstruction. Patients with intestinal obstruction in both groups recovered after conservative treatment with gastrointestinal decompression, fasting with water, and intravenous nutrition. Anastomotic leakage occurred in three patients (4.1%) in the control group, while none occurred in the experimental group. One patient with an anastomotic leak in the control group had a poor, conservative outcome and was discharged after timely ileostomy with a leak drainage tube. Meanwhile, the remaining two patients were cured after conservative treatments, such as abdominal drainage, parenteral nutrition strengthening, and anti-infective drugs. No significant differences in the first postoperative feeding, first exhaustion, first defecation, intestinal obstruction, anastomotic stricture, anastomotic bleeding, postoperative hospital stay tumor stage, first postoperative routine blood test, or tissue typing were observed between the experimental and control groups (*P* > 0.05). The incidence of anastomotic leakage was significantly lower in the experimental group than in the control group (*χ*^2^ = 4.520, *P* = 0.033), as presented in (Table 3).

**Table 3:** Analysis of postoperative factors

	Control group (*n* = 70)	Experimental group (*n* = 77)	Statistical data	*P*-value
First postoperative meal^a^ (d, mean ± SD)	6.46 ± 1.58	6.4 ± 0.73	−0.265	0.792
First postoperative venting^a^ (d, mean ± SD)	5.97 ± 1.44	5.71 ± 0.60	−1.384	0.17
First defecation after operation^a^ (d, mean ± SD)	5.87 ± 1.14	5.79 ± 0.70	−0.502	0.616
Intestinal obstruction^b^ (*n*)			2.564	0.109
Yes	2	1		
No	68	76		
Anastomotic fistula^b^ (*n*)			4.52	0.033
Yes	3	0		
No	67	77		
Anastomotic stenosis^b^ (*n*)			0.028	0.866
Yes	1	1		
No	69	76		
Anastomotic bleeding^b^ (*n*)			2.599	0.107
Yes	2	1		
No	68	76		
Post-operative hospital days^a^	13.11 ± 5.45	11.91 ± 3.22	−1.613	0.11
(d, mean ± SD)				
First postoperative routine^b^				
(mean ± SD)				
Albumin (g/L)	33.65 ± 6.2	33.11 ± 3.76	−0.624	0.534
Hemoglobin (g/L)	108.86 ± 19.9	103.88 ± 17	−1.634	0.104
Red blood cells (1012/L)	3.99 ± 0.65	3.89 ± 0.51	−1.061	0.29
Tissue typing^b^ (*n*)			2.067	0.559
Low differentiation	22	19		
Medium differentiation	34	46		
Highly differentiated	5	7		
Undifferentiated	9	7		

Control group, cross side-to-side anastomosis; experimental group, circular side-to-side anastomosis; *SD* standard deviation

^a^*t*-test

^b^Chi-square test

## Discussion

Since 2008, the National Comprehensive Cancer Network has included radical laparoscopic surgery for colorectal cancer as one of the principles of surgical treatment [16]. In right hemicolectomy, the common anastomoses used in ileocolic surgery are end-lateral anastomosis, lateral anastomosis, and end-to-end anastomosis [17]. Anastomotic leakage, stenosis, and bleeding are common complications of colon cancer [18]. Among them, postoperative anastomotic leakage is the most severe and common complication, which increases the perioperative morbidity and mortality rate of patients, leading to the possibility of permanent stoma and seriously affecting their quality of life [19].

At present, many domestic and foreign experts have still not reached a consensus on the choice of postoperative anastomosis for colon cancer. Baqar et al. [20]. have concluded that in using lateral anastomosis after ileocolic resection when having a more extensive anastomosis, the operation time of lateral anastomosis is shorter, and the procedural steps are more straightforward than that of other anastomoses; however, the incidence of postoperative anastomotic leakage complications was not considered. By contrast, the results of a study by Lee et al. [21] have demonstrated that the end-lateral anastomosis approach was more beneficial for patient recovery, which was attributed to end-lateral anastomosis causing minor damage to the circular smooth muscle of the intestine, earlier recovery of bowel function, and significantly less postoperative bowel obstruction and anastomotic leak complications. However, although the anastomosis was regular in end-lateral anastomosis, the size of the anastomosis was fixed with the diameter of the tubular anastomosis, and patients were prone to a higher risk of postoperative complications due to various factors after resuming feeding than with the other two anastomoses. Chierici et al. [22] concluded that end-to-end anastomosis reduces the incidence of anastomotic fistula in the treatment of laparoscopic low rectal cancer. However, postoperative anastomotic leakage, intestinal obstruction, anastomotic stricture, and diarrhea were more frequent in patients who underwent ileocolic end-to-end anastomosis. In ileocolic end-to-end anastomosis, mesenteric margin-to-mesenteric margin anastomosis is generally used, and the intestinal wall in the mesenteric triangle has no plasma membrane, which does not easily heal and is prone to poor suturing during anastomosis. Due to the low blood supply in the colon, healing may be slightly slower, thus making the colon prone to ischemic necrosis, leading to anastomotic leakage. In a prospective study, Kim et al. [23] have reported no significant difference in each postoperative complication caused by end-lateral and lateral anastomoses. After comparing the various anastomoses, the optimal anastomosis remains undetermined. However, regardless of the anastomosis selected, the risk of anastomotic leakage after laparoscopic-assisted radical right hemicolectomy for right-sided colon cancer remains.

Currently, no consensus has been established regarding the incidence of postoperative complications, such as anastomotic leakage and anastomotic bleeding caused by different anastomotic approaches. The risk factors for anastomotic leakage are multifaceted, and several factors, including the patient’s general condition, preoperative treatment, surgical procedure, prophylactic fistula, and postoperative management, may affect the incidence of postoperative anastomotic leakage [24]. In this experiment, the ileocolic anastomosis in both groups was lateral, and the anastomosis was wide and straight, which could reduce the risk of postoperative anastomotic stricture as well as the speed of intestinal contents passing through the anastomosis so that the contents of the small intestine could be absorbed more fully, and the number of postoperative bowel movements could be reduced. In the experimental group with circular anastomosis, the possibility of stump ischemia caused by two anastomotic staples crossing each other at the common stump of the ileum was effectively avoided. Combined with the analysis of additional patient data, no significant differences in operation time, time to first postoperative expectoration and defecation, or first postoperative blood count were observed between the two groups. For the recovery of gastrointestinal function in postoperative patients, this study assessed recovery of the gastrointestinal tract by observing the time of first postoperative exhaustion and defecation. However, no significant difference in postoperative bowel recovery function was identified between the two types of anastomosis in this study, which may be related to the fact that the damage to the intestinal muscle structure had no significant difference between the two techniques. Therefore, this study demonstrated that the incidence of postoperative anastomotic leakage was significantly different between the two groups, which may be related to the application of circular anastomosis in the experimental group, the absence of anastomotic staple intersections at the ileocolic stump, and the rich blood supply to the stump compared with the control group, which fully demonstrated the advantage of circular lateral anastomosis of the ileocolon in laparoscopic-assisted right hemicolectomy. Based on the data on the occurrence of anastomotic leakage in this study, the incidence of anastomotic leakage was relatively high in the control group (three patients in total) and none in the experimental group. No significant differences in general baseline information and anastomotic leakage impact indicators (e.g., preoperative treatment status, surgical approach, inclusion of prophylactic fistula and postoperative management, and many other factors) were noted between the two groups. Stump blood perfusion is reportedly a critical factor in anastomotic healing in colorectal surgery, regardless of the anastomosis selected [25]. Preoperative and postoperative factors that may cause anastomotic leakage were excluded, and intraoperative factors that caused anastomotic leakage were further analyzed. In conclusion, the anastomoses formed by both circular and cross-lateral anastomoses have many characteristics, although the richness of the blood supply at the anastomoses significantly differed between the two groups. In the control group, the anastomotic staples of the two anastomoses inevitably formed four right-angle ischemic areas after stump closure. Although intraoperative needle embedding is feasible, poor blood supply may occur, which may lead to anastomotic leakage. By contrast, anastomosis in the experimental group effectively avoided crossover of the anastomotic staple, reducing the probability of producing an ischemic area at the anastomosis, which reduced the incidence of postoperative anastomotic leakage. From the data on other postoperative complications in both groups, no significant differences in postoperative complications were observed between the two groups (*P* > 0.05). For the development of postoperative intestinal obstruction, the result of postoperative intestinal obstruction in the animal model experiments is associated with neuroimmune, inflammatory responses, or delayed electrical rhythms [26, 27]. Taking together the general data of this study and the actual situation of the patients, all patients with postoperative obstruction were of advanced age (>60 years), and postoperative obstruction was considered to be related to poor bowel function in advanced age and not directly related to the two anastomosis methods.

In a study on the choice of in vitro or in vivo anastomosis in laparoscopic right semicolon cancer surgery, Małczak et al. [28] collected and analyzed the primary pathological data of clinical patients. In patients undergoing laparoscopic right hemicolectomy, intra-abdominal anastomosis and extra-abdominal anastomosis had different effects on the recovery of intestinal function. In this study, we observed that intestinal recovery was slightly faster with intra-abdominal ileocolonic anastomosis, recovery of peristalsis was faster, and the time to the first defecation was earlier. In a recent large observational study, Anania et al. [29] have reported that intra-abdominal anastomosis was associated with longer operation time. By contrast, a meta-analysis by Selvy et al. [30] has revealed no significant difference in the operation time between intra-abdominal anastomosis and extra-abdominal anastomosis. Total laparoscopic surgery is widely known to be more technically demanding, and the learning curve is steeper than that of open surgery; therefore, laparoscopic surgery requires the surgeon to be more skilled. However, for right hemicolon cancer tumors, intra-abdominal anastomosis and extracorporeal anastomosis require tumor removal in all orifices in the abdomen. Our study involved laparoscopic-assisted right hemicolectomy. Using two different extra-abdominal anastomosis techniques, the information of the two groups of patients was compared to determine their significance in postoperative complications.

Meanwhile, Van Oostendorp [31] analyzed and compared the in vitro and in vitro anastomosis of laparoscopic right hemicolectomy. They suggested that in vivo anastomosis had a lower incidence of postoperative complications and faster patient recovery when performing laparoscopic right hemicolectomy. Moreover, in vivo anastomosis during laparoscopic right hemicolectomy was associated with reduced short-term morbidity and shorter hospital stay, indicating faster patient recovery. They believe that [31] in the choice of abdominal incision location, the middle/upper abdominal incision has a higher incidence of postoperative complications than the incision in the lower abdomen. Since we selected a caudal approach for treating right-sided colon cancer, this position is not very demanding for the transverse colon. We typically selected the upper navel opening, which is approximately 5 cm in size. We considered the different intestinal anatomy of the other patients. Simultaneously, to ensure smooth anastomosis in vitro, the opening location will be determined according to the situation. Our experience was as follows: for the experimental group using circular lateral anastomosis, when performing ileal–colonic stump anastomosis, the ileal stump should be adequately maintained with a common starting point (points I and H) due to the anatomical structure. Stitches were intermittently added to fix the ileocolic stump, lift the assistant, and straighten each fixation line to ensure that the ileocolic stump was on the same level, and the stump was closed with a linear cutting closure below the fixation line to ensure complete anastomosis. Meanwhile, crossover of the two anastomotic staples should be avoided to ensure blood supply to the stump and reduce the occurrence of anastomotic ischemia. At the junction of the anastomosis, it should be reinforced with internal turning and embedding sutures. Circular lateral anastomosis can be used not only for ileal–colonic anastomosis after right hemicolectomy, but also during colonic–colonic anastomosis after radical surgery for tumors in the left hemicolectomy, transverse colon, and proximal sigmoid colon. Moreover, the application of this anastomosis method may be relatively broad, and the operation process is not complex. Thus, it can be used in almost all digestive tract surgeries and provides new ideas for surgeons. Compared with the control method, the blood supply to the stump is more abundant, which can accelerate the recovery of patients. Additionally, this kind of anastomosis is widely used, not only in laparoscopic anastomosis but also in extra-abdominal surgery, even in Da Vinci robotic surgery. However, at present, the circular anastomosis of right-sided colon cancer is mainly performed in vitro, although in Kanaya et al. [32], Japanese scholars have first reported the new technique of complete laparoscopic Bi-I type gastrointestinal anastomosis. Subsequently, studies have been published on complete laparoscopic left hemicolectomy. We believe that performing ileal–colonic loop anastomosis in vivo may be challenging, and the tumor specimen still needs to be removed through the abdominal wall incision postoperatively. Compared with traditional open surgery, circular lateral anastomosis is a relatively new method for right hemicolon anastomosis. However, this study technique has some limitations. First, this technique is too complicated to perform laparoscopically in patients with obesity or mesenteric contractures and may require conversion to open surgery. Second, our medical team opted to clean and ligate the mesenteric blood vessels with high accuracy during laparoscopic right hemicolectomy to ensure the integrity of the intestinal blood supply, which requires high technical requirements for laparoscopic operation and ultrasonic scalpel use. This will also increase the operation time. Nevertheless, the strengths are as follows. First, in patients with ring-side anastomosis, the intestinal anastomosis was wide, and the anastomotic nails were not crossed. Compared to cross anastomosis, ring-side anastomosis can reduce the risk of postoperative complications. Second, this method can be applied to various digestive tract anastomoses, making the operation simple and easy to use.

## Conclusions

In conclusion, in laparoscopic radical right hemicolectomy, the postoperative recovery differs between anastomotic approaches, as demonstrated in various comparisons. This study revealed that circular lateral anastomosis for right hemicolon cancer was superior to cross-lateral anastomosis in reducing the incidence of postoperative anastomotic ischemic area, which deserves further promotion. However, to avoid errors, the included patients were operated on by the same surgical team. Therefore, multicenter, prospective, and large-sample clinical studies are needed to verify this finding.

## Data Availability

Not applicable.

## Abbreviations

ASA: American Society of Anesthesiologists
BMI: Body mass index
MDT: Multidisciplinary team
SD: Standard deviation
